# Epstein Barr Virus-Encoded EBNA1 Interference with MHC Class I Antigen Presentation Reveals a Close Correlation between mRNA Translation Initiation and Antigen Presentation

**DOI:** 10.1371/journal.ppat.1001151

**Published:** 2010-10-14

**Authors:** Sebastien Apcher, Chrysoula Daskalogianni, Benedicte Manoury, Robin Fåhraeus

**Affiliations:** 1 Cibles Thérapeutiques, INSERM U940, Institut de Génétique Moléculaire, Université Paris 7, Hôpital St. Louis, Paris, France; 2 Institute Curie, Paris, France; University of Wisconsin-Madison, United States of America

## Abstract

Viruses are known to employ different strategies to manipulate the major histocompatibility (MHC) class I antigen presentation pathway to avoid recognition of the infected host cell by the immune system. However, viral control of antigen presentation via the processes that supply and select antigenic peptide precursors is yet relatively unknown. The Epstein-Barr virus (EBV)-encoded EBNA1 is expressed in all EBV-infected cells, but the immune system fails to detect and destroy EBV-carrying host cells. This immune evasion has been attributed to the capacity of a Gly-Ala repeat (GAr) within EBNA1 to inhibit MHC class I restricted antigen presentation. Here we demonstrate that suppression of mRNA translation initiation by the GAr in *cis* is sufficient and necessary to prevent presentation of antigenic peptides from mRNAs to which it is fused. Furthermore, we demonstrate a direct correlation between the rate of translation initiation and MHC class I antigen presentation from a certain mRNA. These results support the idea that mRNAs, and not the encoded full length proteins, are used for MHC class I restricted immune surveillance. This offers an additional view on the role of virus-mediated control of mRNA translation initiation and of the mechanisms that control MHC class I restricted antigen presentation in general.

## Author Summary

The presentation of short peptides on major histocompatibility (MHC) class I molecules forms the cornerstone for which the immune system tells apart self from non-self. It is important for viruses such as the Epstein-Barr virus (EBV) to avoid this antigen presentation pathway in order to escape recognition and killing of its host cells. All EBV-infected cells, including cancer cells, express EBNA1 without attracting the attention of the immune system. In this report we describe the mechanism by which EBNA1 escapes antigen presentation. This should open up for new approaches to target EBV-associated diseases including cancers and immuno proliferative disorders and for understanding the underlying mechanisms of the source and regulation of antigenic peptide production.

## Introduction

Presentation of antigenic peptides on major histocompatibility (MHC) class I molecules is a signal for CD8^+^ T cells to distinguish between cells that express self or non-self antigens and forms an important part of the immune system's capacity to fight parasite invasion. There are several steps that endogenous peptides pass from their synthesis to the loading onto the MHC class I molecule. On one hand, the digestion of the peptide precursor by the proteasome [1], [2], [3], the affinity of the peptide for the TAP transporter [4], the trimming of the N-terminus by endopeptidases [5] and the sequence requirements of the peptide to fit the grove on the MHC class I molecules [6], are all important steps in determining the efficiency of peptide presentation. On the other hand, the steps prior to the digestion of the peptide precursor by the proteasome, the so called pre-proteasomal steps, have to ensure that enough peptide material is produced so that a sufficient amount of the correct peptide epitopes reaches the class I molecules in order to trigger a T cell response. It has been estimated that approximately 10^4^–10^5^ MHC class I molecules are expressed by individual cells at any time to ensure a sufficient antigen presentation.

Proteins and polypeptides exhibit a wide range of half-life, with an overall average of 1 to 2 days [7]. As the stability of viral proteins is many times high, it would take many hours for the cells to accumulate a sufficient amount of viral peptides to trigger the most efficient T-cell response if these were derived from the degradation of the full length protein. To explain the rapidity of viral-antigen presentation, a model has been proposed in which a fraction of rapidly degraded mRNA translation products (RDPs) [8] or defective ribosomal products (DRiPs) [9] with a half-life of less than 10 minutes constitute the main source for antigenic peptides. This model has been supported by the rapid slow down of TAP system by blocking protein synthesis and the equal rapid suppression of antigen presentation when transcription of an mRNA encoding a protein with a long half life is shut off [10]. In addition, cryptic mRNA translation products derived from different reading frames throughout the message can provide substrates for the MHC class I pathway [11]. The use of alternative translation products as a source for antigenic peptides, together with the fact that continues ribosomal activity is required for antigen presentation, implicates mRNA translation as an important pre-proteasomal step in regulating MHC class I restricted antigen presentation. However, the translation mechanisms that govern the synthesis of antigenic peptide products are unknown.

Viruses adapt to their environment and manipulate their host cells in order to serve their needs. Controlling the MHC class I antigen presentation pathway is an important target for latent viruses in order to avoid detection of the infected host cell by the immune system. There are many examples of different strategies whereby viral products target the MHC class I pathway on the post-proteasomal level [12], [13] but there is so far little known about how viruses affect the steps that control the production of antigenic peptides. The Epstein-Barr virus (EBV) expresses the nuclear antigen-1 (EBNA1) in all types of infected cells and in its type I latent form, e.g. observed in Burkitt's lymphoma, it is the only viral antigen detected [14]. A Glycine-Alanine repeat sequence (GAr) located in the N-terminal part of EBNA1 with no apparent biochemical function has a *cis*-acting capacity to suppress presentation of antigenic peptides to the MHC class I pathway and plays an important role for the EBV to evade immune detection [15], [16]. Like EBNA1, Kaposi's sarcoma-associated herpesvirus LANA1 protein and the MHV-68 gamma herpes virus ORF73 are latent origin binding proteins that act for maintaining viral episomes in infected cells. These two proteins have more recently been suggested to use a similar strategy, but with different sequences, as EBNA1 to escape the MHC class I pathway [17], [18], indicating that this might be a more commonly used concept among viruses to evade the immune system.

It was recently shown that the nascent GAr peptide targets the initiation step of translation of any mRNA to which it is fused [19]. Here we have used GAr-mediated control of mRNA translation initiation to study its effect on MHC class I restricted antigen presentation. By manipulating the GAr sequence we can control the rate of translation initiation of a reporter mRNA in *cis* and we can thereby demonstrate that the rate of translation initiation, as opposed to other means of translation control, directly determines the amount of presented peptides derived from the main open reading frame as well as from cryptic translation products of a given mRNA. We discuss how these results fit together with the concept of EBNA1 as an immunologically silent protein and the proposed models for the source of antigenic peptide material for the MHC class I pathway.

## Results

### Direct influence of a Gly-Ala repeat on the presentation of a reporter epitope

**Figure 1:**
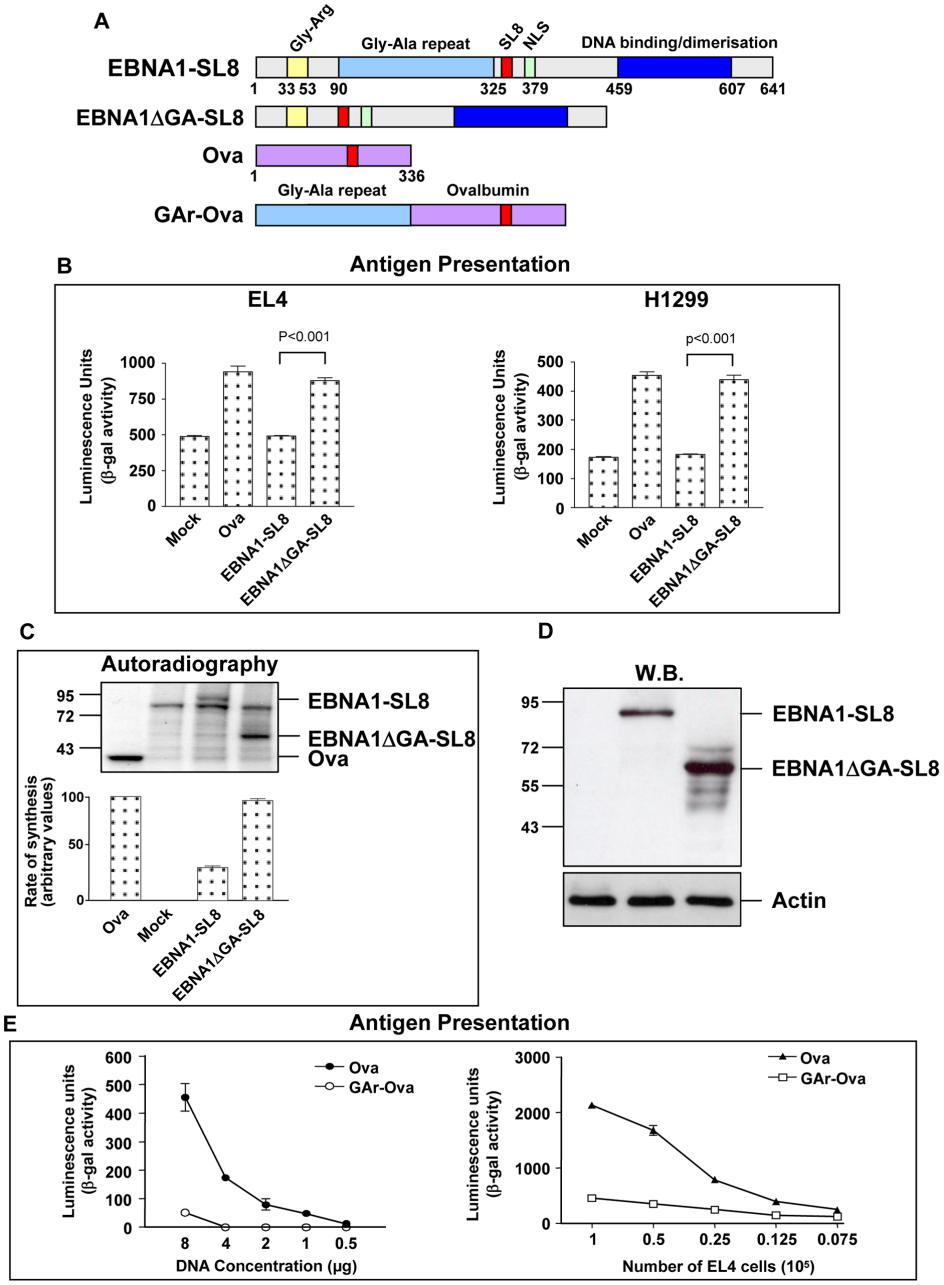
Inhibition of EBNA1 synthesis prevents presentation of peptides derived from the EBNA1 mRNA. A) Cartoon illustrating the different constructs. The location of the exogenous antigenic peptide sequence SIINFEKL (SL8) of the chicken ovalbumin (Ova) in the EBNA1-SL8 and the EBNA1ΔGA-SL8 constructs is indicated. B) The presentation of SL8 peptide on endogenous MHC class I K^b^ molecules on (0.5×10^5^) EL4 cells (left) or on human cells co-expressing a genomic K^b^ construct (right) was determined using B3Z CD8^+^ T cells [23]. The GAr domain suppresses presentation of SL8 by over 90% in either cell type. C) Autoradiograph of a 1 hour ^35^S-methionine pulse label in the presence of proteasome inhibitors shows that the EBNA1-SL8 mRNA is translated 60% less efficiently as compared with the EBNA1ΔGA-SL8. The graph below shows values determined from phosphoimager analysis. D) Western blot shows the steady state level of expression of indicated constructs without proteasome inhibitors. E) Dose-response curve shows that approximately 8 µg of GAr-Ova cDNA is required to reach a similar level of SL8 presentation as that of 1 µg of Ova (left panel). Increasing number of EL4 cells expressing indicated constructs in the presence of a fixed amount (5×10^4^) of B3Z (right graph). The results show representative data from at least three independent experiments in which transfected cells were split and tested for protein synthesis or antigen presentation with SD.

The Gly-Ala repeat sequence (GAr) of EBNA1 can prevent mRNA translation initiation and MHC class I restricted antigen presentation from open reading frames (ORF) to which it is fused [19], [20]. To test to which degree the GAr is responsible for the suppression of EBNA1 antigen presentation we fused the SIINFEKL encoding antigenic peptide sequence (SL8) derived from chicken Ovalbumin (Ova) [21], [22] into the EBNA1 (EBNA1-SL8) or in an EBNA1 in which the Gly-Ala repeat (GAr) was deleted (EBNA1ΔGA-SL8) (Fig. 1A). This allowed us to monitor any effect the GAr has on antigen presentation from these mRNAs using the B3Z CD8^+^ T hybridoma that is specific for the SL8 in the context of H-2K^b^ MHC class I molecules [23].

H-2K^b^ EL4 cells expressing Ova or EBNA1ΔGA-SL8 gave a similar level of presentation of the SL8 epitope, demonstrating that there is no significant difference in how this antigen is processed and presented between these two constructs. However, presentation of SL8 in the context of EBNA1 was dramatically suppressed and comparable to cells expressing an empty vector (Fig. 1B, left graph). Similar results were obtained using the H1299 human cell line in which a vector coding the mouse H-2K^b^ MHC class I molecule was cotransfected together with the expression vectors for Ova, EBNA1-SL8 or EBNA1ΔGA-SL8 respectively (Fig. 1B, right graph).

To test how this difference in antigen presentation correlates with GAr's inhibitory effect on protein synthesis, H1299 cells expressing each construct were pulsed for 1 h in the presence of ^35^S-methionine and proteasome inhibitor in order to minimize any effects of proteasomal degradation before harvested. This revealed that EBNA1-SL8 is translated with approximately 60% reduced efficiency as compared to the EBNA1ΔGA-SL8 (Fig. 1C). Western blot analysis showed that the steady state protein expressions of the respective proteins correlate with their respective rate of synthesis (Fig. 1D).

Similar results in terms of control of synthesis and antigen presentation were also obtained when the GAr sequence was fused to the N-terminus of Ova itself. Fusion of the full length GAr to the N-terminus of ovalbumin (GAr-Ova) effectively prevents the presentation of the SL8 peptide over a wide range of mRNA concentrations and eight times the amount of a GAr-Ova cDNA was required to reach the same level of antigen presentation as from cells expressing Ova alone (Fig. 1E, left panel). To ensure that the antigen presentation reporter system was not saturated under these conditions we increased the number of Ova expressing EL4 cells that were exposed to the same fixed number (5×10^4^) of B3Z cells used in the above experiments (Fig. 1E, right panel).

These results, together with previous reports, collectively support the notion that the GAr domain alone inhibits presentation of peptides to the MHC class I pathway from the EBNA1 or from any mRNA to which it is fused, irrespectively of location [19], [20].

### The GAr suppresses presentation of antigenic peptides throughout the entire mRNA

It is known that the GAr in addition to preventing translation initiation also has the capacity to inhibit protein unfolding and proteasome-mediated degradation in a substrate- and position-dependent fashion [24]. In order to investigate if the capacity of the GAr to affect protein stability plays a role in its capacity to provide immune evasion we separated its two functions.

**Figure 2:**
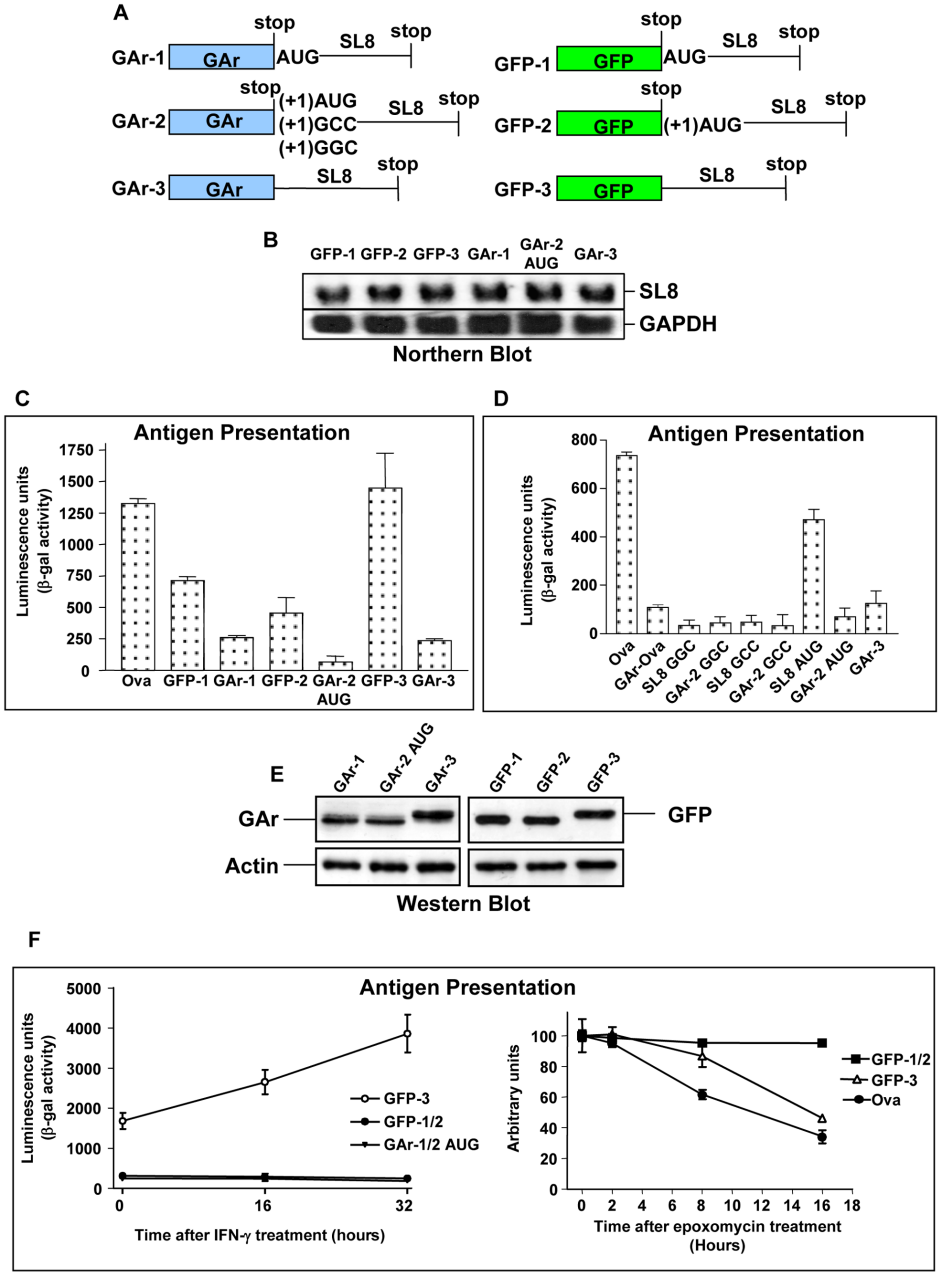
Inhibition of protein degradation is not essential for the GAr sequence to prevent endogenous antigen presentation for the MHC class I restricted pathway. A) The SL8 epitope was inserted in the 3′UTR of the GAr open reading frame (ORF) (GAr-1), in another reading frame (GAr-2) or fused to GAr (GAr-3). The corresponding constructs were also made in which the SL8 was inserted in the GFP mRNA in an identical way (GFP-1 to 3). The AUG, GCC or GGC initiation codons for SL8 in GAr-2 were used. B) Northern blot analysis using the SL8 sequence as probe shows that the GAr sequence does not influence the expression levels of the corresponding mRNAs. C) The GAr suppresses presentation of SL8 throughout the entire mRNA, demonstrating that its capacity to prevent antigen presentation does not depend on controlling protein degradation. D) Changing the initiation codon of the SL8 from AUG to GGC (Glycine) or GCC (Alanine) in the GAr-2 prevents expression and antigen presentation, demonstrating that the SL8 is derived from an individual translation initiation event. Presentation of SL8 expressed as a minigene using the initiation codons AUG, GGC and GCC (SL8AUG, SL8GGC and SL8GCC, respectively). E) Western blot shows the steady state level of expression of indicated constructs (GAr-1 to 3 and GFP-1 to 3). F) IFNγ stimulates antigen presentation by optimising processing of peptides longer than 8–10 residues and has only an effect on antigen presentation when SL8 is expressed in the main ORF and not when expressed as cryptic peptide in an alternative reading frame (left graph). Epoxomicin is a specific proteasome inhibitor and prevents presentation of SL8 when expressed in the main ORF (right graph). This shows that expression of the SL8 epitope as a cryptic peptide does not carry additional residues from the main reading frame. Data are representative of three or more independent experiments and values are shown with SD. Protein synthesis and antigen presentation data are derived from cells transfected with the indicated constructs before split and tested separately.

The SL8 was inserted in the 3′ untranslated region (UTR) of the GAr sequence, either in the same or in a different reading frame (GAr-1 and GAr-2, respectively) where it is expressed as a cryptic minigene [25], [26]. Thus, any effect the GAr has on antigen presentation from these mRNAs can be separated from its capacity to inhibit proteasomal degradation since the GAr and the SL8 epitope are expressed as separate polypeptides. We also fused the SL8 peptide in frame with the C-terminus of the GAr (GAr-3) (Fig. 2A). In addition, we made the corresponding constructs where we exchanged the GAr sequence for that of the GFP (GFP-1, GFP-2 and GFP-3 respectively) (Fig. 2A). The GFP is a suitable replacement for the GAr as it is also a protein with a low turnover rate, a poor substrate for the proteasomes [27] and, importantly, the levels of mRNAs expressing the SL8 are similar in the context of either ORF (Fig. 2B). These different constructs allowed us to compare the effect of the GAr on suppressing MHC class I antigen presentation independently of its capacity to influence protein degradation. The level of presentation of SL8 from the GFP-3 is similar to that from Ova itself, demonstrating that there is no significant difference in how the antigen is processed in these two settings. However, presentation of the SL8 in the context of all GAr-carrying mRNAs is suppressed as compared with the corresponding GFP constructs, demonstrating that the GAr prevents mRNA antigen presentation throughout the entire mRNA (Fig. 2C). The GFP-1 and GFP-2 constructs give a lower level of presentation of the SL8 compared to when fused to the C-terminus of GFP (GFP-3) (Fig. 2C). This is explained by the fact that the antigenic peptide in GFP-1 and 2 are expressed as cryptic translation products compared with when it is fused to the main reading frame in GFP-3 and Ova. To ensure that the expression of SL8 from the GFP-2 and GAr-2 minigene constructs are indeed derived from an initiation event and not from a read-through from the main ORF we substituted the AUG codon with GGC or GCC. As this completely prevented antigen expression from either constructs it shows that the expression of the SL8 is not due to a read-through event and, thus, that the GAr suppresses a reinitiating event (Fig. 2D). Western blot analysis confirms that the expression levels from the main ORF of the different constructs are similar (Fig. 2E). The notion that SL8 expressed from the 3′UTR is derived from an independent initiation event is further supported by treating cells with IFNγ. IFNγ stimulates the induction of immunoproteasomes and N-terminal trimming peptidases that together give a more efficient processing of peptides longer than 8–10 residues for loading onto MHC class I molecules [28], [29]. IFNγ treatment does not affect presentation of the SL8 when inserted in the 3′UTR, which is what is expected if it is expressed as a minigene, and only when fused directly to the C-terminus of GFP or GAr (Fig. 2F, left panel). By treating cells with the proteasome inhibitor epoxomicin we observed that that presentation of SL8 is proteasome-dependent when derived from Ova, or fused to GFP, but not when translated as an out-of-frame minigene downstream of GFP (GFP-1 &-2) (Fig. 2F, right panel) or GAr (GAr-1 &-2) (data not shown). These results show that the cryptic translated SL8 peptides are derived from independent translation initiation and do not carry additional residues from the main upstream reading frame that could interfere with the processing of the MHC class I peptide.

Taken together, these results show that the GAr suppresses the presentation of the SL8 epitope within the same reading frame and out-of-frame epitopes. Hence, the GAr suppresses MHC class I restricted antigen presentation by preventing translation initiation throughout the entire mRNA and its potential capacity to control protein stability is not required to impose immune evasion.

### Altering mRNA translation initiation overrides the effect of the GAr

**Figure 3:**
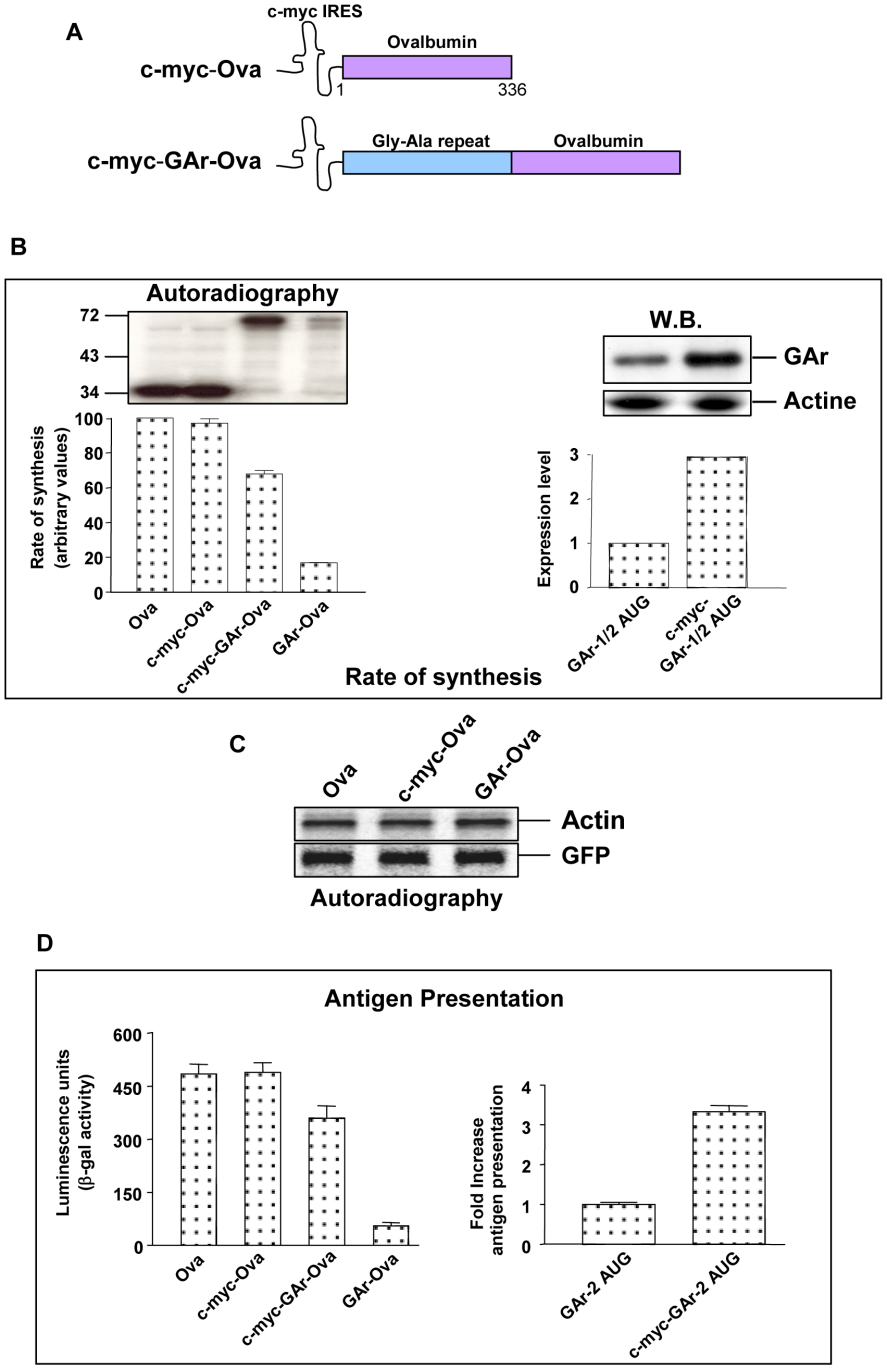
GAr suppresses antigen presentation by targeting the mRNA translation initiation process. A) Cartoon illustrating the c-myc Internal Ribosomal Entry Sites (IRES) constructs. IRESs offer alternative cap-independent mechanisms of mRNA translation initiation. B) Autoradiograph of ^35^S-methionine metabolic pulse labelling. Insertion of the c-myc IRES in the 5′UTR of the GAr-Ova mRNA (c-myc-GAr-Ova) restores translation in H1299 cells but does not affect translation when inserted in the 5′UTR of Ova alone (left panel). Western blot shows that the effect of the c-myc IRES is restricted to the GAr alone (right panel). C) Autoradiograph of a 30 minutes ^35^S-metabolic pulse label experiment of the endogenous protein (actin) and the exogenous GFP protein in H1299 cells in the presence of the c-myc IRES and the GAr constructs. D) The c-myc IRES stimulates SL8 presentation in the context of the GAr from the main open reading frame as well as cryptic translated products (see Fig. 2A). Data are representative of three or more independent experiments plus SD.

The nascent GAr peptide is regulating the synthesis of EBNA1 by directly blocking initiation of the EBNA1 mRNA translation in *cis* which is caused by a delay in the assembly of the initiation complex [19]. The molecular target of the GAr is not yet known but we have observed that insertion of the c-myc IRES [30] in the 5′UTR of GAr-Ova (c-myc-GAr-Ova) overrides GAr-dependent inhibition of protein synthesis and restores the rate of expression to approximately 70% of that of Ova alone (Figs. 3A and 3B, left panel). Similarly, the c-myc IRES also induced the expression of the GAr alone approximately 3-fold, demonstrating that this effect is restricted to the GAr itself (Fig. 3B, right panel). The c-myc IRES has no effect on the rate of Ova synthesis when inserted in an identical way in the 5′UTR (Fig. 3B, left panel), indicating its specific effect on GAr-mediated translation control. Moreover, the c-myc IRES and the GAr domain only affect mRNA translation in *cis* as we do not see any changes on the rate of translation of actin or of the exogenous GFP (Fig. 3C).

Thus, the combination of the GAr and the c-myc IRES provides us with tools with which we can study the relationship between the rate of mRNA translation initiation and the production of antigenic peptides from a single mRNA without targeting protein synthesis or degradation using general chemical inhibitors. When we tested the effect of the c-myc IRES on GAr-dependent control of antigen presentation we observed a 70% presentation of SL8, as compared to Ova alone or c-myc-Ova (Fig. 3D). Under the same conditions the presentation of SL8 from the GAr-Ova fusion construct was approximately 5-fold less. As the c-myc IRES does not affect the GAr-Ova ORF this result further underlines that the potential effect of the GAr to control the stability of the protein to which it is fused is not sufficient to suppress antigen presentation. Insertion of the c-myc IRES in the 5′UTR of the GAr-2 mRNAs also resulted in a sharp increase in antigen presentation, demonstrating that the same mechanism of translation initiation control that regulates the production of antigenic peptides derived from the main ORF also regulates the production peptides derived from cryptic minigenes.

The capacity of the c-myc IRES to neutralise the translation inhibitory effect of the GAr is cell specific and was observed in three out of three human cell lines tested (Table 1). However, it has been shown that the efficiency of the c-myc IRES-driven translation varies between cell lines from different origins. In murine cell lines the c-myc IRES-driven translation is much lower than in human cell lines and importantly it has been shown to be inactive in murine adult tissue [31], [32]. This explains the finding that the c-myc IRES was incapable to override the translation inhibitory effect of the GAr in all the murine cell lines tested (Table 1).

**Table 1:** GAr-dependent inhibition of antigen presentation in different cell lines from different origins.

Cell line	Cell Origin	(%) antigen presentation as compared to Ova or EBNA1ΔGA		(%) antigen presentation as compared to Ova
		GAr-Ova	EBNA1	c-myc-GAr-Ova
H1299	Human Lung carcinoma	15±0.8	5±1.1	70±5.8
Saos-2	Human osteosarcoma	15±1.5	N.T.	70±6.3
EL4	Mouse lymphoma	8±1.1	3±0.8	8±2.8
B6	Mouse fibroblast	20±1.4	5±1.6	20±2.5
Ramos	Human Burkitt's lymphoma	2±0.3	2±1.1	63±3.3
NIH3T3	Mouse embryonic fibroblast	15±1.8	20±2.3	15±3.4
B16F10	Murine Melanoma	5±1.7	5±2.1	5±1.9

The presentation of SIINFEKL from chicken ovalbumin (Ova) itself or Ova inserted in the EBNA1 coding sequence was detected using the B3Z reporter cells. The presentation of SIINFEKL from Ova or from an EBNA1 construct that lacks the GAr sequences (EBNA1ΔGA) was given the arbitrary value of 100%. The right column shows the effect of the c-myc IRES on GAr-dependent inhibition of antigen presentation in cell lines from different origins. The table shows data from at least three experiments and SD. The c-myc IRES overrides GAr-dependent antigen presentation in human derived cell lines only. (N.T.  =  not tested).

**Figure 4:**
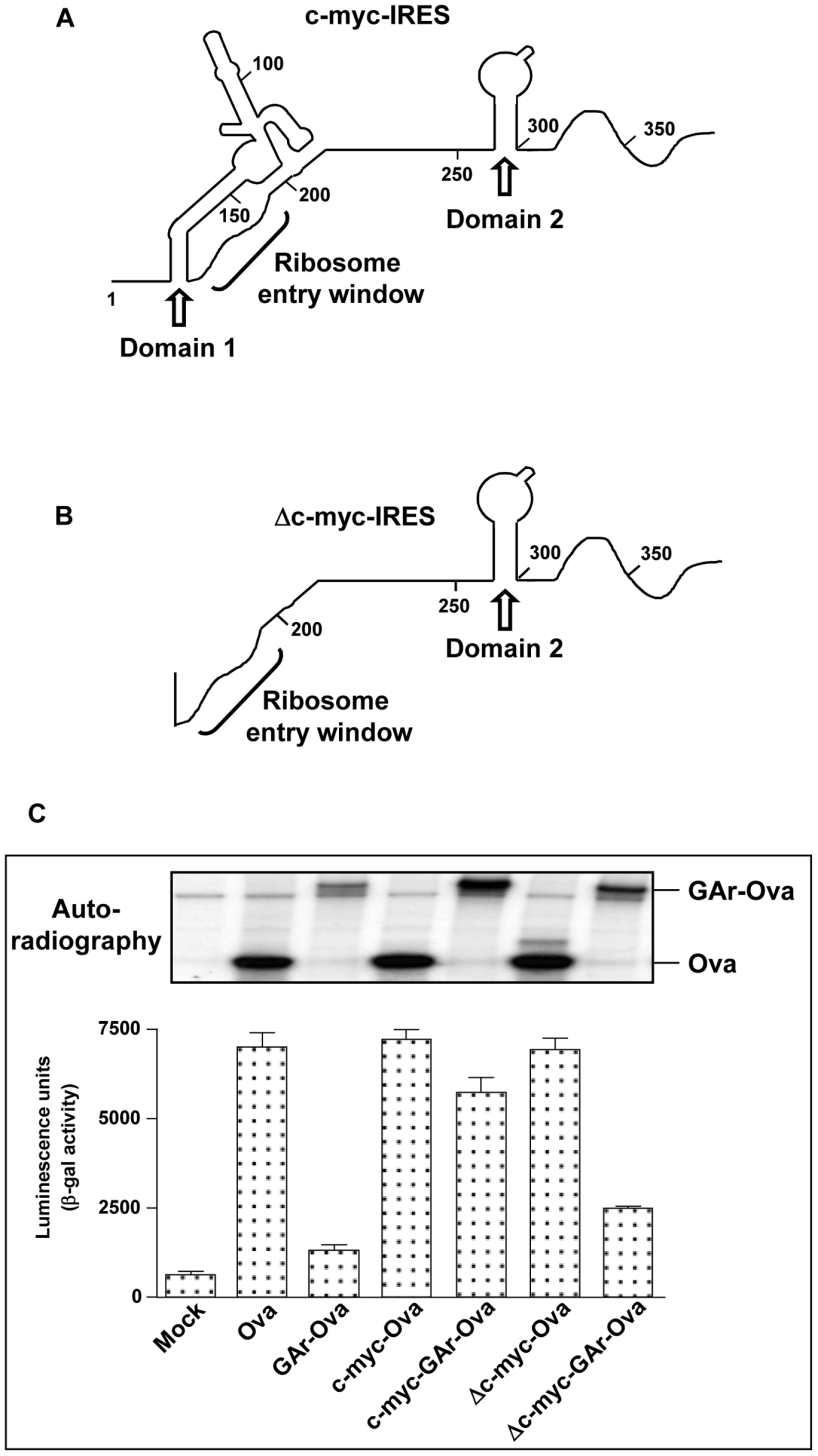
Domain 1 of the c-myc IRES is responsible for the effect of the c-myc IRES on GAr-dependent translation control. A) Cartoons illustrating the predicted structure and functional domains of the human c-myc IRES and domains 1 and 2 and the ribosome entry window are indicated. B) Domain 1 was deleted while retaining the ribosome entry window and domain 2 (Δc-myc IRES). C) Autoradiograph of ^35^S-methionine metabolic pulse labelling and presentation of SL8 derived from the indicated constructs in H1299 cells. Insertion of the Δc-myc IRES in the 5′UTR of the GAr-Ova mRNA (Δc-myc-GAr-Ova) does not restore translation as compare to the intact c-myc IRES (c-myc-GAr-Ova). Neither Δc-myc IRES nor c-myc IRES affect translation when inserted in the 5′UTR of Ova alone. Data are representative of three or more independent experiments with SD.

The c-myc IRES has been characterised and consists of different domains and predicted ribosome entry window (Figs. 4A and 4B). It has been shown that deletion of the domain 1 reduces its activity with about 60% [33]. In line with this, fusion of a c-myc IRES, that lacks domain 1 (Δcmyc-IRES), in the 5′UTR of Ova-GAr results in a reduced capacity to override suppression of translation and antigen presentation (Fig. 4C). This further links the effect of the c-myc IRES and its capacity to overcome GAr-dependent suppression with its capacity to control mRNA translation initiation (Fig. 4C).

These results show that GAr-mediated suppression of translation initiation is sufficient and necessary to prevent antigen presentation from the main ORF as well as from cryptic translation products and that its effect can be neutralised by alternative mechanisms of initiation provided by the c-myc IRES.

### The rate of mRNA translation initiation directly determines antigen presentation

**Figure 5:**
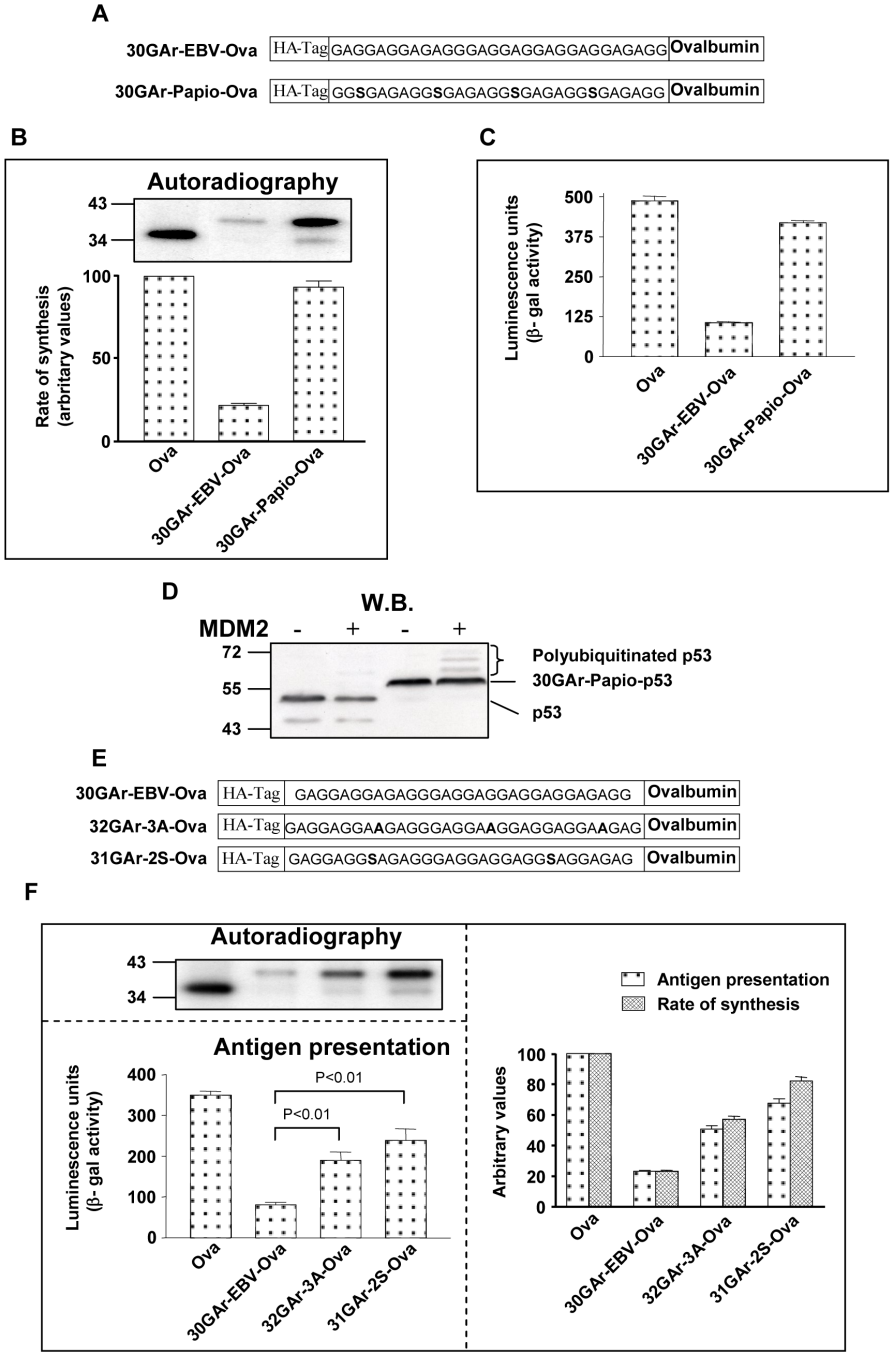
The rate of mRNA translation initiation directly correlates with the amount of antigen presented from a given mRNA. A) A 30 amino acid GAr sequence from the EBV-encoded EBNA1was fused to the N-terminus of Ova (30GAr-EBV-Ova). The GAr sequence from the EBNA1-like protein of the Papio virus carries four single inserted serine residues (30GAr-Papio-Ova). B) Autoradiograph of a 30 minutes ^35^S-methionine pulse label. C) Presentation of SL8 derived from corresponding constructs in EL4 cells. D) The p53 protein is targeted for the ubiquitin-dependent degradation pathway by the E3 ligase MDM2 [42]. Fusion of the Papio GAr to p53 results in an accumulation of polyubiquitinated products in the presence of MDM2, showing that the Papio GAr retains the capacity to affect protein degradation [24]. E) The GAr sequence consists of single alanines separated by one, two or three glycines. Introducing 2 alanines (GCC) next to each other on 3 separate places (32GAr-3A-Ova) does not alter the overall GC content of the RNA sequence. F) Introducing a single serine next to an alanine at two locations (31GAr-2S-Ova) is more disruptive in terms of mRNA translation as compared with the 32GAr-3A-Ova (left, upper panel). The corresponding effect on antigen presentation is shown in the graph below. The right graph shows the arbitrary values of the rate of mRNA translation initiation and antigen presentation. Data are representative of three or more independent experiments and values are shown with SD. Cells were transfected with the indicated constructs before split and tested separately for antigen presentation or synthesis.

The observations that the increase in rate of synthesis after insertion of the c-myc-IRES corresponds to antigen presentation indicates a close correlation between the rate of mRNA translation initiation and the capacity of CD8^+^ T cells to detect and destroy virus-infected host cells. In order to look more closely at this relationship, we wanted to change the rate of Ova synthesis more subtly compared with the “on/off” effect obtained with the c-myc-IRES and we used mutated GAr sequences that have been shown to affect the synthesis of GAr fusion proteins. The lymphocrypto-Papio and the Rhesus viruses infect Old World primates and express EBNA1 homologues that carry shorter GAr-like sequences that have previously been shown not to prevent antigen presentation [34]. The EBV-GAr sequence consists of single alanine residues separated by one, two or three glycines while the Papio-GAr carries single serine residues inserted in every seven residues of the repeat (Fig. 5A and [19]). When we fused a 30 amino acid Papio-GAr sequence (30GAr-Papio-Ova) and a corresponding 30 amino acid EBV-GAr sequence (30GAr-EBV-Ova) to the N-terminus of Ova we observed that the Papio-GAr-like sequence had no effect on mRNA translation or antigen presentation while insertion of the corresponding EBV-GAr sequence resulted in an approximately 4-fold less synthesis and antigen presentation (Figs. 5B and 5C). Similar results were also obtained with the Rhesus GAr-like sequence which also carries serine insertions (data not shown). Interestingly, the Papio-GAr has the capacity to control protein stability and fusion of the Papio-GAr to the p53 protein, which is normally targeted for the ubiquitin-dependent degradation pathway by the MDM2 E3 ligase, resulted in a stabilisation and in an accumulation of polyubiquitinated 30GAr-Papio-p53 products in the presence of MDM2 (Fig. 5D). This indicates that while the Papio sequence retains the effect on proteins stability, the disruption of its GAr sequence is sufficient to render it inefficient in preventing antigen presentation or protein synthesis control [24].

We next tested a construct in which the GAr repeat had been disrupted by inserting two alanines next to each other on three locations (32GAr3A-Ova) (Fig. 5E). This retains the GC rich content of the GAr RNA sequence without introducing new amino acid residues. In this case, the rate of synthesis was approximately 50% less compared with Ova alone but over two-fold more efficient than that of the wild type GAr (30GAr-EBV-Ova) (Fig. 5F, left panel). If two glycine residues were instead replaced by serines (31GAr2S-Ova) we obtained a 75% translation efficiency as compared to Ova alone. When we next compared the effects of these GAr sequences on antigen presentation we observed that the rate of mRNA translation initiation is closely followed by the amount of antigens presented to the MHC class I molecules (Fig. 5F, right panel).

Taken together, these results indicate a direct and proportional relationship between endogenous antigen presentation and mRNA translation initiation control.

## Discussion

Our results further underline the notion that the capacity of EBNA1 to evade the MHC class I antigen presentation pathway and the detection by CD8^+^ T cells relies on the Glycine-Alanine repeat (GAr) sequence [15], [16], [20], [35]. Deletion of the GAr sequence from EBNA1-SL8 resulted in the same amount of antigen presentation as when SL8 was presented from the Ova message indicating that no other regions of EBNA1 are needed to evade MHC class I antigen presentation. Dose-response experiments show that this effect is not dependent on the amount of mRNA expressed in the cells and that at least eight times the amount of a GAr-carrying mRNA is required in order to reach a similar level of antigen presentation as that of a corresponding non-GAr carrying mRNA.

**Figure 6:**
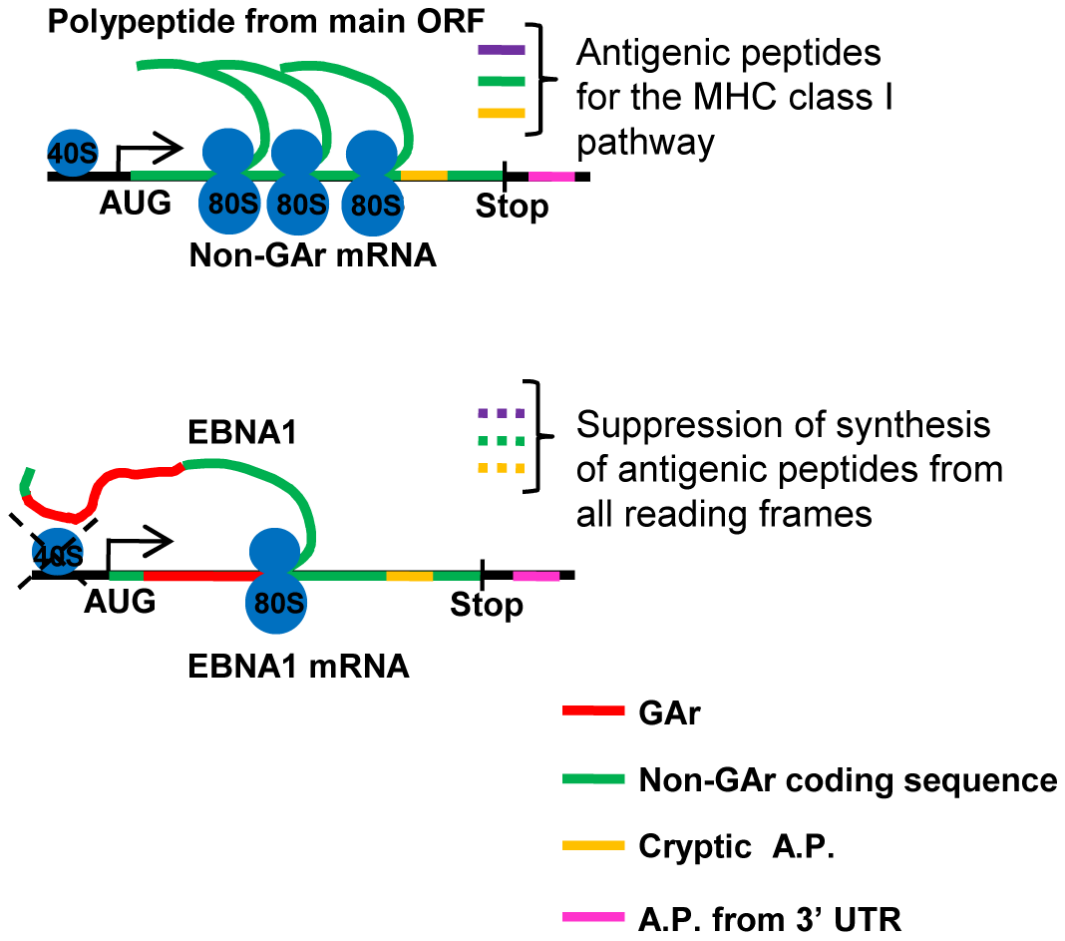
Antigenic peptides (A.P.) can be derived from the main open reading frame as well as cryptic peptides from alternative reading frames (yellow) and from the 3′UTR (pink) [**25**]. The nascent GAr polypeptide (red) of the EBNA1 prevents translation initiation throughout the entire mRNA, including its own reading frame and cryptic peptides. This allows the EBV to evade the MHC class I restricted antigen presentation of peptides from the EBNA1 message and helps the virus to evade the immune system. The GAr also prevents the synthesis of the EBNA1 full length protein but its long half life ensures that functional levels of EBNA1 are expressed.

The GAr has the unique dual capacity to suppress both its own mRNA translation initiation as well as the stability of proteins to which it is fused. By separating these two functions from each other we have shown that the GAr suppresses peptide production from different reading frames of an mRNA to which it is fused and, hence, that control of mRNA translation initiation is both sufficient and necessary for its capacity to suppress MHC class I antigen presentation (Fig. 6). Previous studies have shown that the GAr can prevent unfolding of substrates targeted for the 26S proteasome by affecting 19S-dependent unfolding in a substrate and position-dependent fashion. However, the GAr has no, or little, effect on the stability of EBNA1 itself, suggesting that fusion of GAr to 26S proteasome substrates gives unspecific effects on protein stability that are unlikely to play any physiological role for the virus [24]. These observations together with our results instead support a model in which the *cis*-mediated effect of the GAr on EBNA1 mRNA translation initiation is the sole mechanism by which EBNA1-expressing latently EBV-infected cells can evade recognition by the immune system. However, this does not mean that EBNA1 stability is not an important feature in EBV's strategy to evade the immune system for the simple reason that a low rate of EBNA1 synthesis requires a low turnover rate in order to allow a sufficient amount of EBNA1 to be expressed (Fig. 6). The expression of EBNA1 in the host resting B memory cells, as compared to rapidly proliferating BL cells, is likely less, which could further contribute to help the virus to establish an immune evasive latency.

Several results lead us to propose that control of initiation of mRNA translation, and not prevention of elongation, is the key feature for the GAr domain in controlling MHC class I antigen presentation. Firstly, the insertion of the c-myc IRES in the 5′UTR of GAr-carrying mRNAs prevents the GAr from suppressing mRNA translation and antigen presentation. It is unlikely that the insertion of an IRES in the 5′UTR without any changes to the main reading frame could impose differences in GAr-dependent control of synthesis other than via altering the initiation conditions. This is further supported by the observations that deletion of the domain 1 of the c-myc IRES prevents its effect on overcoming the GAr and its cell line-dependent mode of action. This might also indicate that the target factor of the GAr is independently recruited to the polysome by domain I and could further help to elucidate the mechanisms of GAr action on translation initiation. Secondly, the GAr-like sequence derived from the Papio virus, where a single serine residue is inserted at every seven amino acids, has little effect on the rate of protein synthesis or on antigen presentation. Furthermore, by inserting three alanines into the EBV-GAr sequence we retain the GC rich mRNA sequence but increase synthesis and antigen presentation. Finally, the GAr suppresses presentation of antigenic peptides that are derived from independent translation initiation events in the 3′UTR of the GAr-encoding reading frame. The latter type of translation initiation of cryptic minigenes was shown by the group of Shastri to be sufficient to provide antigenic peptides for the MHC class I pathway [25]. It is difficult to see how pre-termination of the translation due to difficulties for the ribosome in reading through the GC rich region could explain suppression of independent down-stream initiation events [36]. In addition, truncated EBNA1 peptides due to failure of elongation are not observed in EBV infected cells. Taken together, neither of the observations presented here are likely to occur if the GAr acts via mechanisms related to elongation, including difficult ribosomal read-through, codon exhaustion, or other more specific mechanisms [36], [37]. Previous studies have shown that changes to the GAr peptide sequence, but not RNA sequence impair its efficiency to suppress translation [19], underlining that the effect is mediated by the peptide, and not the RNA sequence. The GAr is predicted to be unstructured and does not included charged residues and, as expected, a 30 amino acid GAr peptide was found not to bind the EBNA1 mRNA ([20] and data not shown). However, a recent report suggests that the Gly-Arg repeat of EBNA1 has RNA binding capacity [38].

Blocking translation initiation offers an explanation to how the GAr can succeed in suppressing production of DRiPs/RDPs and thus antigenic peptides derived from initiation events from all reading frames throughout the mRNA. Based on the model that antigenic peptides are not derived from degradation of full length proteins it should not be possible for the GAr to avoid presentation of peptides derived from EBNA1 by controlling its turnover rate since this would only affect peptides derived from the full length EBNA1 and not DRiPs [39] or RDP products that do not carry the GAr. Our previous work and toeprint analysis carried out by others indicate that the GAr peptide has to be synthesised in order to suppress mRNA translation initiation and that it does not affect the site of initiation [19], [36]. This is line with the notion that the GAr would not give rise to truncated EBNA1 peptides due to alternative initiation sites or diffuse pre-termination events that would not serve the function of the protein and thereby not support viral replication, nor prevent presentation of upstream antigenic peptides, but to an overall suppression of translation *in cis*. In fact, using GAr specific polyclonal sera one does not see any massive accumulation of truncated EBNA1 products in normal EBV-infected cells that would have been expected from a pre-termination event derived from within the GAr sequence.

One question that arises from this study is if this mechanism of evading the immune system is efficient, it should be adapted by other viruses. It has recently been suggested that the MHV-68 gamma herpes virus ORF73 is using a similar mechanism as EBNA1 to evade MHC class I restricted antigen presentation. In this case, however, the sequence is not identical to the GAr, suggesting that other amino acid sequences can achieve a similar effect [17]. The LANA-1 of Kaposi's sarcoma virus is also believed to use a similar mechanism but with a different repeat sequence [18]. Hence, several viruses might use a similar concept but different sequences. This makes it more difficult to predict how wide spread this type of mRNA translation control is among viruses but it indicates that the strategy to escape the MHC class I pathway by manipulating mRNA translation initiation applies to several viruses and is not restricted to the EBV.

The GAr acts in *cis* and offers a unique opportunity to study the relationship between antigen presentation, protein stability and mRNA translation without the addition of general chemical inhibitors of protein synthesis or degradation that might have indirect or unspecific effects. By making minimal changes to the GAr amino acid sequence we have shown that we can fine tune translation initiation of the mRNAs and, as far as we are aware, there are no other systems described that allow this. The GAr consists of single alanines disrupted by one, two or three glycines. Disruption of the GAr by two alanines next to each other on three locations is sufficient to reduce the translation and antigen presentation inhibitory effect of 30 amino acids GAr sequence by approximately 50%. Introduction of two serines on two locations has a more disruptive effect and reduces its effect with approximately 75%. This demonstrates a close correlation between the rate of mRNA translation initiation and MHC class I restricted antigen presentation that has consequences for understanding the source of antigenic peptides for the MHC class I pathway. These results are in line with other studies suggesting that protein stability does not affect antigen presentation [40] and indicates that a fundamental part of the immune system's capacity to detect virus-infected host cells is reliant on the mechanism of viral mRNA translation, as opposed to any features linked to the actual viral proteins. This supports the notion that MHC class I restricted immune surveillance is in fact directly correlated with the mechanisms that regulate protein synthesis and not protein degradation and supports the model where it is in fact the presence of mRNA, and not the full length proteins, that is surveilled by the MHC class I pathway [41]. This opens up for novel ways of interpreting viral control of mRNA translation and new approaches for therapeutic intervention aimed at virus associated diseases. These results also have broader implications in the understanding of the peptide selection process and will allow the prediction of antigenic peptide production from specific mRNAs that has implications for generating more efficient DNA vaccines and potentially also for better understanding of dysregulated antigen presentation in autoimmune disease and the generation of self tolerance.

## Methods

### Cell culture and transfection

The murine lymphoma cell line EL4 (H-2K^b^) and B3Z cell line were maintained in complete medium, consisting of RPMI-1640 medium supplemented with 10% heat-inactivated fetal calf serum (FCS), 25 mM Hepes-buffer solution (Gibco-BRL, Santa Clara, CA), 100 IU/ml penicillin and streptomycin (Gibco-BRL), 2 mM L-glutamine (Gibco-BRL), 2 mM sodium pyruvate solution (Gibco-BRL), 2 mM non-essential amino acid solution (Gibco-BRL), and 0.5 µM of 2-β mercaptoethanol (2-βME) (Sigma-Aldrich, St. Louis, MO). Transient transfections of EL4 cells were performed by electroporation using a BMX pulser Bio-Rad Gene Pulser II (Bio Rad, Hercules, CA) at 260 mV in a 0.4 cm Bio-Rad electroporation cuvette. EL4 cells were washed once in washing medium, consisting of RPMI medium 1640 supplemented with 2% heat-inactivated fetal calf serum (FCS), 25 mM Hepes-buffer solution (Gibco-BRL), 100 units/ml penicillin, and and 100 µg/ml streptomycin (Gibco-BRL), 2 mM L-glutamine (Gibco-BRL), 2 mM sodium pyruvate solution (Gibco-BRL), 2 mM non-essential amino acid solution (Gibco-BRL), and 0.5 µM of 2-βME (Sigma Chemical). The cells were then suspended in complete medium at a concentration of 7.5×10^6^/ml. 8 µg of plasmid were inoculated with 3×10^6^ cells in the electroporation cuvette. Immediately after electroporation, the cells were transferred to a 6-well plate with 3 ml of complete media. Human cell lines were cultivated under standard conditions in RPMI medium 1640 (H1299 and Saos-2), each containing 10% FCS, 2 mM L-glutamine, 100 units/ml penicillin, and 100 µg/ml streptomycin. Cells were seeded in 6-well plates at a density of 1.75×10^5^ cells/well. The following day the cells were cotransfected with 1 µg total of expression plasmids along with 3 µl of Genejuice according to the manufacturer's protocol (Merck Biosciences, Darmstadt, Germany).

### Electrophoresis and western blotting

Following separation on 12% SDS-PAGE, proteins were transferred to 0.45 µm nitrocellulose membranes, and blots were blocked for 1 hour at room temperature with a 5% skim milk in TBS solution consisting of 20 mM Tris, 500 mM NaCl, 0.1% Tween 20, pH 7.5. Blots were incubated overnight at 4°C with anti-EBNA1 mouse monoclonal antibody (OT1X) (1∶1000) or polyclonal anti-GA antibody (1∶500), raised against the Gly-Ala sequence of EBNA1 protein or a monoclonal actin antibody (1∶1000) Chemicon International (Temecula, CA) or anti-p53 rabbit polyclonal antibody (CM-1). The membranes were washed before incubated with horseradish peroxidase-conjugated rabbit anti-mouse or mouse anti-rabbit immunoglobulin antibody (1∶5000) for another 1 h and detected using ECL (Amersham Bioscience). The ECL signal was quantified using CCD camera and associated software (Vilber Lourimat, France). Pre-stained molecular markers were from Fermenta (Ontario, Canada).

### Plasmid constructions

All plasmids were generated using standard procedures. Restriction enzymes, T4 DNA ligase and calf intestinal alkaline phosphatase were obtained from New England Biolabs (Ipswich, MA). Purified synthetic oligonucleotides were obtained either from MWG biotech (Ebersberg, Germany) or Eurogentec. Routine plasmid maintenance was carried out in DH5α and TOP10 bacteria strains.

The EBNA1 and EBNA1ΔGA were generated using oligonucleotide pairs 5′AGTATAATCAACTTTGAAAAACTCTGAGAAG3′ and 5′CTTCTCAGAGTTTTTCAAAGTTGATTATACT3′, encoding the SL8 peptide, inserted into the unique Bstx1 site found in EBNA1 sequence, right after the GAr sequence.

The GFP-1 construct was prepared using oligonucleotide pairs 5′**AATTC**TGAATGAGTATAATCAACTTTGAAAAACTCTGA**T**3′ and 5′**CTAGA**TCAGAGTTTTTCAAAGTTGATTATACTCATTCA**G**3′, encoding the SL8 peptide, inserted into the EcoR1/Xba1 sites of EGFP-C2 vector (BD Biosciences Clontech, Palo Alto, CA). The GAr-1 construct was made using the same oligonucleotide pairs inserted in the 3′UTR of the GAr, itself cloned into the pCDNA-3 vector (Invitrogen, Carlsbad, CA). The GFP-2 and GAr-2 constructs were made in the same way using the oligonucleotide pairs 5′**AATTC**CTGAATGAGTATAATCAACTTTGAAAAACTCTGA**T**3′ and 5′**CTAGA**TCAGAGTTTTTCAAAGTTGATTATACTCATTCAG**G**3′. The different GAr-2 constructs were prepared by mutating the AUG codon in GCC or GGC using standard procedures. The GFP-3 and GAr-3 constructs were prepared using the oligonucleotide pairs 5′**AATTC**AGTATAATCAACTTTGAAAAACTCTGA**T**3′and 5′**CTAGA**TCAGAGTTTTTCAAAGTTGATTATACT**G**3′ and inserted in the same vectors.

The c-myc IRES cDNA was obtained from Dr. A.C. Prats (INSERM U589, France). The pCDNA3-Ova and pCDNA3-GAr-Ova constructs were obtained as described previously [20]. c-myc-Ova, c-myc-GAr-Ova and c-myc-GAr-2 were generated by amplification of full length human c-myc IRES by polymerase chain reaction (PCR), using a 5′ sense primer containing a BamH1 site 5′C**GGATCC**ACTAGAACTCGCTGTAGTAATTC3′ and a 3′ antisense primer 5′TCC**GGATCC**GCGGGAGGCTGCTGG 3′ containing another BamH1 site. The fragment was cloned into the 5′UTR digested pCDNA3-Ova, pCDNA3-GAr-Ova and pCDNA3-GAr-2 constructs.

The 30GAr-EBV-Ova construct was made by replacing the full-length GAr sequence in the Gar-Ova construct with an oligonucleotide sequences corresponding to 30 amino acids of the GAr. The same approach was used to produce 32GAr3A-Ova, 31GAr2S-Ova and 30GAr-Papio-Ova.

### Metabolic cell labeling and immunoprecipitation

All mRNA translation assays were carried out in H1299 cells transfected with indicated constructs. Transfected cells were cultured for 36 hours before treated with 20 µM MG132 for one hour in methionine free medium containing 2% dialysed FCS. 0.15 mCi/ml of [^35^S] methionine (Perkin Elmer, Boston, USA) was added in the presence of proteasome inhibitor and the cells were harvested at indicated time points using a rubber policeman after 2x washing in cold PBS. Cell pellets were snap frozen at −80°C before lysed in PBS containing 1% NP40 and Complete protease inhibitor cocktail (Roche Diagnostics GmbH, Mannheim, Germany) at 4°C. Lysates were centrifuged for 15 minutes at 14.000 rpm and pre-cleared by addition of mouse sera and protein G sepharose. An equal amount of total protein was incubated with specific antibodies for 4 hours at 4°C before the immune complexes were recovered using protein G sepharose. The proteins were separated on precast Bis-Tris 4–12% SDS-PAGE (Invitrogen) and the amount of labelled protein was visualized by autoradiography and the relative amount of protein synthesis was determined using phosphoimager.

### T-cell assay

EL4-Kb restricted cells (5×10^4^) expressing the indicated constructs for 48 h were washed in medium and cultured with 5×10^4^ B3Z T cell hybridoma for at least 20 h in 96-well plates. T cell assays in human H1299 cell lines were done by co-transfecting the Kb expression vector together with the reporter construct. The B3Z CD8^+^ T cell hybridoma expresses LacZ in response to activation of T cell receptors specific for the SIINFEKL peptide (OVA-immunodominant peptide) in the context of H-2K^b^ MHC class I molecules. The cells were then harvested and washed 2 times with 1X cold PBS prior to lysis in 0.2% TritonX-100, 0.5M K_2_HPO_4_, 0.5M KH_2_PO_4_ for 5 min on ice. The lysates were centrifuged for 10 min and 25 µl of supernatant from each well were transferred into 96-well optiplate counting plates (Packard Bioscience, Randburg, SA). The plates were incubated for 1 hour at room temperature, protected from light and tested for β-Galactosidase activity using the Luminescence assay (BD Biosciences Clontech) on a FLUOstar OPTIMA (BMG LABTECH Gmbh, Offenburg, Germany). The results were expressed in counts per seconds (CPS) or in relative light units (RLU). The peptides SIINFEKL (corresponding to ovalbumin amino acids 258–276) was purchased from Eurogentec (Seraing, Belgium).

### Northern blotting analysis

Total RNA was isolated from EL4 cells. After separation on agarose gels, the RNA was transferred to nylon filter (Hybond-N+; Amersham Bioscience). An oligonucleotide probe corresponding to the SIINFEKL sequence was previously labelled with [^32^P]ATP using the Ready-to-Go kit (Amersham Bioscience). After baking the membrane at 80°C for 2 h, the RNA was hybridized overnight with the probe at 42°C in PerfectHyb Hybridization Solution (TOYOBO, Tokyo, Japan). The membranes were washed twice for 5 min in 1× sodium chloride/sodium citrate (SSC : 0.15 M NaCl, 15 mM sodium citrate, pH 7.0) that contained 0.1% SDS at room temperature and twice for 1 h in 1×SSC that contained 0.1% SDS at 55°C. The RNA was visualized by autoradiography.
